# Evidence that climate sets the lower elevation range limit in a high‐elevation endemic salamander

**DOI:** 10.1002/ece3.4198

**Published:** 2018-07-06

**Authors:** Evan H. Campbell Grant, Adrianne B. Brand, Stephan F. J. De Wekker, Temple R. Lee, John E. B. Wofford

**Affiliations:** ^1^ SO Conte Anadromous Fish Laboratory USGS Patuxent Wildlife Research Center Turners Falls Massachusetts; ^2^ Department of Environmental Sciences University of Virginia Charlottesville Virginia; ^3^ Shenandoah National Park Luray Virginia

**Keywords:** climate change, cloud base height, competition, co‐occurrence, occupancy, range limits

## Abstract

A frequent assumption in ecology is that biotic interactions are more important than abiotic factors in determining lower elevational range limits (i.e., the “warm edge” of a species distribution). However, for species with narrow environmental tolerances, theory suggests the presence of a strong environmental gradient can lead to persistence, even in the presence of competition. The relative importance of biotic and abiotic factors is rarely considered together, although understanding when one exerts a dominant influence on controlling range limits may be crucial to predicting extinction risk under future climate conditions. We sampled multiple transects spanning the elevational range limit of *Plethodon shenandoah* and site and climate covariates were recorded. A two‐species conditional occupancy model, accommodating heterogeneity in detection probability, was used to relate variation in occupancy with environmental and habitat conditions. Regional climate data were combined with datalogger observations to estimate the cloud base heights and to project future climate change impacts on cloud elevations across the survey area. By simultaneously accounting for species’ interactions and habitat variables, we find that elevation, not competition, is strongly correlated with the lower elevation range boundary, which had been presumed to be restricted mainly as a result of competitive interactions with a congener. Because the lower elevational range limit is sensitive to climate variables, projected climate change across its high‐elevation habitats will directly affect the species’ distribution. Testing assumptions of factors that set species range limits should use models which accommodate detection biases.

## 1 INTRODUCTION

Understanding the controls on species’ range limits is a central topic in ecology and evolution. Range limits may result from species interactions in the absence of strong environmental variation (Price & Kirkpatrick, 2009), or under steep spatial gradients in environmental conditions (Case, Holt, Mcpeek, & Keitt, 2005), which may allow persistence of populations with narrow physiologic limits (Hampe & Jump, 2011). A frequent assumption is that biotic interactions set the range limit at the lower elevation bounds of a species distribution (the “warm edge”; Davis, Jenkinson, Lawton, Shorrocks, & Wood, 1998; Pearson & Dawson, 2003), while climate controls the upper elevation limit (MacArthur, 1972; Parmesan et al., 2005), although there are few empirical tests of this hypothesis (Cahill et al., 2014; Wilson et al., 2005). The idea that range limits result from exclusively one mechanism may be a false dichotomy, as multiple mechanisms can strongly influence a species.

Climate change is expected to increase the extinction risk for high‐elevation species by affecting the availability of suitable environmental conditions, especially those with small ranges (Dirnböck, Essl, & Rabitsch, 2011; Ohlemüller et al., 2008). Recent observed elevational increases in range limits for several taxa have been correlated with elevational warming trends (Chen, Hill, Ohlemüller, Roy, & Thomas, 2011; Lenoir, Gégout, Marquet, de Ruffray, & Brisse, 2008; Moritz et al., 2008; Tayleur et al., 2015; Walls, 2009; Wilson et al., 2005). In large part, temperature alone is assumed to be a dominant control of the elevational limits of species and is thus used to model range shifts (Bernardo & Spotila, 2006; Forero‐Medina, Joppa, & Pimm, 2011; Forero‐Medina, Terborgh, Socolar, & Pimm, 2011; Thomas et al., 2004), although species‐level variation in rates of elevational change may be determined by other intrinsic and extrinsic variables (Chen et al., 2011). A range of models link climate and species’ occurrence data to forecast increased future extinction risk of high‐elevation species (Forero‐Medina, Joppa, et al., 2011; La Sorte & Jetz, 2010; Lawler et al., 2009) although some models predict species’ persistence where microrefugia exist in warming habitats (Dobrowski, 2011; Randin et al., 2009; Scheffers, Edwards, Diesmos, Williams, & Evans, 2013). The accuracy of these predictions is dependent on the relative importance of climate variables and species interactions for generating range limits (Pearson & Dawson, 2003).

Elevation is a strong correlate for species’ occupancy in general (Lomolino, 2001; MacArthur, 1972), and for *Plethodon* salamander communities in particular, there are elevational gradients in patterns of diversity (Kozak & Wiens, 2010), species’ interactions (Hairston, 1949, 1951), abundance (Bailey, Simons, & Pollock, 2012), and body size (Hairston, 1949). While elevation is a major factor in defining distributional patterns of eastern *Plethodon*, local habitat factors (which may be independent of elevation) also influence patterns of distribution and mediate the outcome of species’ interactions (Jaeger, 1971a; Rissler, Barber, & Wilbur, 2000). *Plethodon* communities, especially those restricted to high elevations, are expected to be particularly sensitive under current predictions of future climate change (Bernardo & Spotila, 2006; Walls, 2009) because of their thermal and hydric physiologic limits (Spotila, 1972). Both temperature and relative humidity control distribution patterns for salamanders in the genus *Plethodon* (Bernardo & Spotila, 2006; Kozak & Wiens, 2010), which rely on cutaneous moisture for respiration and whose activity is related to temporal and spatial patterns of cool and moist microhabitats (Feder, 1983). Typical of this family of salamanders, the federally endangered Shenandoah salamander (*Plethodon shenandoah*; Highton & Worthington, 1967) is thought to be restricted to talus habitat on elevations above 900 m along the western slopes of the Blue Ridge Mountains in Shenandoah National Park (Jaeger, 1980); if this lower distribution limit is determined by climate variables, it is likely to be unstable given climate change forecasts (Richardson, Denny, Siccama, & Lee, 2003; Wake & Vredenburg, 2008; Walls, 2009). Unlike more widespread species, the small range of this species may afford little chance for local adaptation as there would be little variation in response to climate across the range (Rehm et al., 2015).

The species is similar to other range‐restricted, high‐elevation Appalachian salamanders in that competitive interactions with the congeneric eastern red‐backed salamander (*Plethodon cinereus*) are believed to be the primary determinant of range boundaries (Highton, 1972). Previous studies have investigated multiple hypotheses for competition between these species, but support for a primary mechanism of competition has not been found (Griffis & Jaeger, 1998; reviewed in Jaeger, Gollman, Anthony, Gabor, & Kohn, 2016). It is noted that research on other montane salamander interactions has described climate constraints on physiology as a more important determinant of the lower elevational limit (Arif et al., 2007; Gifford & Kozak, 2012), calling into question the role of competition at this particular part of the range boundary.

In mountain systems, the relationship between atmospheric temperature and elevation in saturated and unsaturated air is well understood. As the pressure and temperature in unsaturated air decrease during ascent, the relative humidity of the air increases to a maximum of 100%, at which point the air becomes saturated and clouds form. This elevation is the cloud base height (CBH), which we hypothesize may be a significant climatic factor affecting the suitability of mountain habitats for occupancy of Plethodon salamanders.

Complicating the observation of true species range limits is the issue of detection biases (Lawton, 1993; Tingley & Beissinger, 2009). There are two related processes that influence the observation of a range edge: an ecological process where a population responds to a biotic or abiotic gradient, and a statistical process where variation in abundance of individuals across space is only partially observed (e.g., Grant, 2014). Ignoring the issue of partial observability may induce bias in the detection of range limits and species interactions, as the presence of one species may influence both the detection and the occurrence of another (Richmond, Hines, & Beissinger, 2010).

While there are theoretical models on the causes of range limits, there are sparse empirical data with which to test these generalizations, and those data that exist may suffer from biases induced by observation errors. Here, we test predictions about the major biotic (competition) and abiotic (climate) factors that govern *P. shenandoah* lower elevational range limits. Many other high‐elevation endemic salamander species are also described as having a distinct lower elevational distribution limit, and therefore, climate sensitivities may likewise be important in defining range limits for high‐elevation salamander communities. Our study provides an operational framework to assess the relative contribution of abiotic and biotic factors on elevational range limits of high‐elevation endemic species.

## 2 METHODS

### 2.1 Site selection and salamander sampling

We turned natural cover (rocks and logs >6 cm in smallest dimension) to find salamanders within 51 sites, with each site located every 100 m along three elevational transects spanning the elevations 700–1,100 m mean sea level (msl) on each of the two highest peaks (Hawksbill and Stony Man) in Shenandoah National Park, VA, USA. A site consisted of two perpendicular 50 × 2 m sampling areas, representing two “spatial replicate” observations (MacKenzie & Royle, 2005). Transects started within the known range of *P. shenandoah* and were spaced ~350–500 m apart running downslope on each mountain. Daytime sampling for salamander occurrence was conducted once for each transect from high to low elevation during September–October 2011 (during which time surface activity of both species was expected to be maximized (Jaeger, 1980). While three species of *Plethodon* may occur in the study area, we primarily detected *P. shenandoah* and *P. cinereus*; the white‐spotted salamander (*P. cylindraceus*) was rarely encountered. We recorded detections of *P. shenandoah* and *P. cinereus* within each spatial replicate.

**Figure 1:**
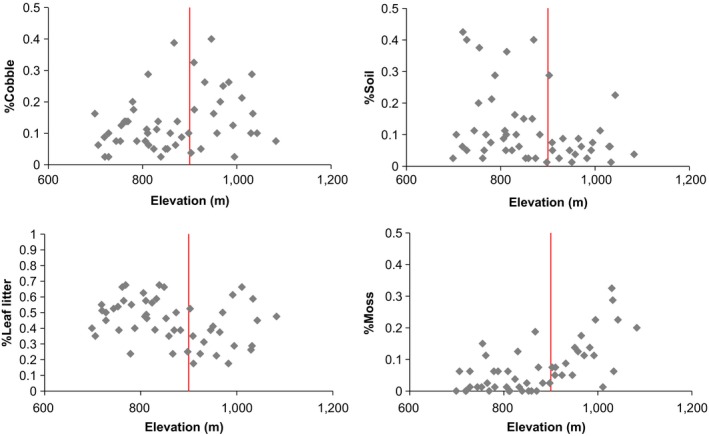
Relationship between elevation (900 m indicated by the vertical red line) and covariates collected at sampling locations along the six elevation transects. Values represent relative percent surface cover of the sampled area (50 m × 2 m) of each category: leaf litter, cobble (diameter between 60 and 256 mm), soil, and moss

We collected covariate data to test the relationship between habitat characteristics and site occupancy of salamanders, including litter and soil depths (in mm), percent cover (soil, moss, cobble [diameter between 60 and 256 mm]), elevation, and aspect. We averaged the soil and litter depth from the beginning, middle, and end of each replicate and visually estimated the percent of each transect covered by soil, leaf litter, cobble, and moss (large woody debris and boulder were correlated with other variables and not considered in the analysis). Most of the covariates are known to be related to the local distribution of these species and are used to delineate talus “types” in earlier work (Jaeger, 1970, 1971b). Talus types are categorical combinations of continuous habitat covariates (cobble, soil, leaf litter), and rather than condense habitat covariates into talus types, we analyzed the habitat covariates directly. Our transects sampled through and beyond areas of talus as mapped by both the National Park Service and the surficial geology map of Southworth et al. (2009); habitat covariates indicative of talus were present beyond the previously defined lower elevation of *P. shenandoah* (Figure 1). Elevation was the average elevation (in meters above mean sea level [msl]) of the start and end point of each spatial replicate (standardized to have mean = 0 and variance = 1 for analysis). We fit a piecewise linear regression model to estimate the breakpoint elevation (Toms & Lesperance, 2003) at which the relationship between percent moss cover and elevation changed (no other habitat covariate showed a threshold change at any elevation, Figure 1).

To determine the relationship between elevation, temperature and relative humidity in and around the habitat of *P. shenandoah*, we deployed Onset HOBO ProV2 temperature–humidity loggers (temperature accuracy: ±0.2°C; relative humidity accuracy: 2.5% between 10% and 90%) in April 2011. Nine loggers were deployed at elevations ranging from 700 to 1,100 m msl along the western side of the Park along slopes with a northerly aspect. Sensors were attached to a fence post, installed 1.5 m above ground level, and enclosed within a radiation shield (Onset‐ RS‐3). The loggers sampled at a frequency of 1 Hz, and 10‐min averages of temperature and relative humidity were recorded.

### 2.2 Statistical models

We fit conditional two‐species occupancy models (Richmond et al., 2010), which estimates the probability of occupancy for a subordinate species conditional upon the presence of a dominant species, which allow for differences in occupancy and detection probabilities of *P. shenandoah* conditional on the presence and/or detection of *P. cinereus*. We tested models which represented whether the occupancy of *P. shenandoah* was conditional [ψ^SC^ different than ψ^Sc^] or unconditional [ψ^S^; i.e., setting ψ^SC^ = ψ^Sc^] on the presence of *P. cinereus* [ψ^C^] and whether occupancy of either species was influenced by site covariates. We evaluated support [via AICc and model weights (Burnham & Anderson, 1956)] for models which examined both additive and interactive effects of mountain and elevation on occupancy for both species. Because of the large number of parameters in the conditional occupancy models, we fit a reasonable a priori set of models via a two‐step process. First, holding the occupancy parameters at a general model structure, we fit 4 different model structures with combinations of detection probability as conditional (or not) upon the occupancy status of each species: (a) Detection of both species was different, but independent of the occupancy state of the other species (pA = rA, pB = rBA = rBa), (b) detection of *P. cinereus* was different and unconditional on the presence of *P. shenandoah*, while the detection of *P. shenandoah* was dependent on occupancy of *P. cinereus* (pA = rA, pB ≠ rBA = rBa), (c) both species had the same detection probabilities when the other was absent, but detection of each species was different when the other species was present (pA = pB, rA ≠ rBA), and (d) both species had different detection probabilities, which differed depending on the presence or absence of the other species (pA ≠ rA, pB ≠ rBA ≠ rBa). We also tested whether the mountain and elevation influenced detection of either species by fitting an additive effect of mountain and elevation which affected both species’ detection probabilities identically. We expected species to have different conditional detection rates, and overall, rates would differ by elevation and mountain.

Using the best‐supported model for detection probability, we then evaluated support for 16 models (Table 1) reflecting biological hypotheses about effects of elevation, mountain, and microhabitat. To evaluate the importance of competition, we ran each model twice: once with occupancy of *P. shenandoah* conditional on *P. cinereus* occupancy [ψ^SC^ ≠ ψ^Sc^], and once where the species occurrences were independent and unconditional [ψ^SC ^= ψ^Sc^ (=ψ^S^)]. We fit models with elevation by species interactions, where *P. shenandoah* occupancy was modeled as either conditional or unconditional on the presence of *P. cinereus*. We also fit models with mountain by species interactions, where *P. shenandoah* occupancy was modeled as unconditional on the presence of *P. cinereus*. We also fit additive models that included combinations of covariates and species’ interactions. Talus‐associated microhabitat covariates were fit with unconditional and conditional models and were assumed to affect each species differently (but not the conditional probability of *P. shenandoah* occupancy). We also included models with a quadratic relationship between salamander occupancy and elevation to allow for a peak in occupancy at an intermediate elevation. We used Akaike's information criterion (adjusted for small sample size; AICc; Burnham & Anderson, 1956) to select the most parsimonious model(s) from our candidate model set. Akaike model weights (*w*_*i*_) were calculated for each model, which represent the weight of evidence for a given model (conditional on the model set; Burnham & Anderson, 1956). As AIC is an index which penalizes (by two times the number of parameters) a model for increasing complexity, in order to be considered informative, the addition of a covariate must result in a likelihood with a difference greater than 2, otherwise it should be ignored (Arnold, 2014). Models were therefore excluded from consideration when the addition of parameters did not result in a change in likelihood (Arnold, 2014).

**Table 1:** Candidate model set used to test whether the occupancy of *Plethodon shenandoah* was conditional [ψ^SC^, ψ^Sc^] or unconditional [ψ^S^; indicating ψ^SC^ = ψ^Sc^] on the presence of *Plethodon cinereus* [ψ^C^] and whether occupancy of either species was influenced by site covariates. We specified covariate effects as conditional [sp(c); indicating a different effect of the covariate on *P. shenandoah* depending on the occupancy status of *P. cinereus*] or unconditional [sp(u); indicating a single effect on *P. shenandoah*] on the presence of *P. cinereus*. Elevation (elev; continuous) and mountain (mtn; categorical with Hawksbill = 1) were included as covariates, and % soil (soil, continuous), cobble (cob, continuous), and leaf litter (leaf, continuous) were included to indicate the presence of talus; constant models were also fit that did not include these covariates. ΔAICc, difference in AIC_c_ value for a particular model when compared with the top‐ranked model; *w*_*i*_, AIC_c_ model weight; *K*, number of parameters in the model; −2LL, twice the negative log‐likelihood value. (Detection structure for all models was {p^A=B^, r^A^, r^BA=Ba^ [elev, isol]}.) Models above the line represent the 95% confidence set (∑*w*_*i*_ **> 0.95)

Model	ΔAICc	*w*_*i*_	*K*	−2LL
ψ^C^ ψ^S^ [elev × sp(u), mtn × sp(u)]	0	0.44	11	152.13
ψ^C^ ψ^Sc^ ψ^SC^ [elev × sp(u), mtn × sp(u)]	2.17	0.15	12	152.3
ψ^C^ ψ^Sc^ ψ^SC^ [elev × sp(u), mtn]	2.67	0.11	11	154.8
ψ^C^ ψ^S^ [elev × sp(u), mtn]	3.08	0.09	10	157.21
ψ^C^ ψ^S^ [elev × sp(u)]	3.75	0.07	9	159.88
ψ^C^ ψ^Sc^ ψ^SC^ [soil × sp(u), cob × sp(u), leaf × sp(u)]	4.21	0.05	16	146.34
ψ^C^ ψ^S^ [soil × sp(u), cob × sp(u), leaf × sp(u)]	5.5	0.03	15	149.63
ψ^C^ ψ^Sc^ ψ^SC^ [elev × sp(u))]	5.63	0.03	10	159.76
ψ^C^ ψ^Sc^ ψ^SC^ [elev × sp(c)]	5.68	0.03	11	157.81
ψ^C^ ψ^S^ [elev, mtn]	8.66	0.01	9	164.79
ψ^C^ ψ^S^ [elev]	12.42	0.00	8	170.55
ψ^C^ ψ^Sc^ ψ^SC^ [elev]	14.42	0.00	9	170.55
ψ^C^ ψ^Sc^ ψ^SC^ [constant]	29.81	0.00	8	187.94
ψ^C^ ψ^S^ [constant]	32.45	0.00	7	192.58
ψ^C^ ψ^S^ [mtn × sp(u)]	35.67	0.00	9	191.8
ψ^C^ ψ^Sc^ ψ^SC^ [mtn × sp(u)]	39.19	0.00	9	195.32

### 2.3 Climate–elevation relationships

We made inference to the CBH elevation (the lower elevation at which clouds form) in the region using three separate data sources. First, to investigate the cloud base elevation in the region, we obtained direct measurements of CBH from the National Climate Data Center for Luray Caverns Airport, located about 13 km west of the *P. shenandoah* habitats. These measurements were made using laser ceilometers that provide CBH at 30‐m height resolution. Second, we summarized relative humidity data from the logger network described above to estimate the location of cloud cover along the western slope. Third, we used a high resolution gridded climate dataset (DAYMET; http://daymet.ornl.gov/custom_home) to investigate how relative humidity changes along the west slope of the Blue Ridge at a near‐constant elevation (900 m msl) extending in a transect between 38.5°N and 38.65°N (~25 km), which encompassed the salamander sampling locations. DAYMET estimates daily meteorological variables, including maximum and minimum temperatures and vapor pressure at a 1 km spatial resolution at a timescale of 1 day from 1980 to 2012. We analyzed the mean relative humidity changes from south to north and calculated the Pearson correlation coefficient between relative humidity and latitude.

Using surface meteorological observations, we then calculated potential future CBHs following Bolton (2015), assuming no change in specific humidity, by applying the temperature change obtained from regional climate models from NARCCAP (http://www.narccap.ucar.edu/; accessed 2014‐10‐27) for the area to present‐day temperatures.

## 3 RESULTS

**Figure 2:**
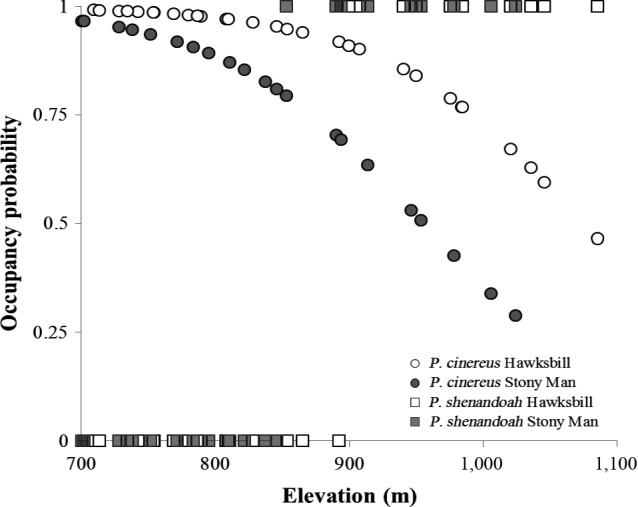
The relationship between elevation and salamander occupancy along the six elevation transects (from the top‐ranked model, ΔAICc = 0; the top 4 models produce near‐equivalent results; Table 1). Circles are *Plethodon cinereus*, squares are *Plethodon shenandoah*; filled symbols are sites on Stony Man, and open symbols are sites on Hawksbill

As expected, the red‐backed and the Shenandoah salamander were the only species encountered; *Plethodon cylindraceus* is known to be present within the Park but is detected very infrequently and was not detected during our surveys. The best‐supported detection model did not differ by species (pA = B), but did differ depending on whether the other species occupied a site, supported higher detection on Stony Man, and supported increasing detection with elevation (with the effect of mountain and elevation the same for both species; r^A^, [r^BA^ = r^Ba^] [elev, isol]). We estimated a near‐zero probability that *P. shenandoah* occupied a site on Stony Man mountain below 850 m, or on Hawksbill mountain below 900 m (Figure 2). In contrast, *P. cinereus* occupancy declined with increasing elevation, without a clear change in distribution around 900 m. The decline in *P. cinereus* occupancy with increasing elevation was more pronounced on Stony Man where the elevational limit for *P. shenandoah* was lower, suggesting that the elevational limits differ among mountains. Contrary to expectations, relative support for competition was weak; across the model set, conditional occupancy models received less than half of the summed AICc weights (∑*w*_*i*_ **= 0.368 for models with ψ^C^ ψ^Sc^ ψ^SC^, Table 1). The model with the greatest support (*w*_*i*_ **= 0.44; Table 1) did not specify a conditional dependence on the presence of *P. cinereus*. It is noted that the second‐best model, which differed from the top model by a difference in AIC_c_ of 2.17, contains only one additional parameter (that which specifies a conditional occupancy of *P. shenandoah*).

**Figure 3:**
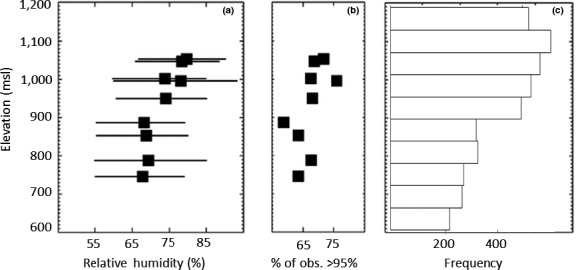
Observations of relative humidity (a), occurrences of cloud cover as indicated by relative humidity >95% (b), and frequency of observed cloud base elevations (c). Whiskers in (a) extend from 25th to 75th percentiles, and filled squares indicate median relative humidity. Data from 1 May 2011 to 30 April 2012 (from dataloggers; panels a, b) and 1 May 2010 to 29 December 2012 (from ceilometer 13 km W of study area; panel c)

Climatological features correlate with the abrupt range limit for *P. shenandoah*, which showed a breakpoint in probability of occupancy around 900 m. While temperature followed the average tropospheric lapse rate (~6.5C/km; Moore, 1956), relative humidity was constant below about 900 m (with median daytime relative humidity values around 75%), but increased by about 10% above ~900 m, despite substantial variability (Figure 3a). The frequency of relative humidity above 95%, indicative of cloud cover (Pick, 1931), also increased above ~900 m (Figure 3b), and this pattern was confirmed by the empirical observations of CBHs measured using the laser ceilometer (Figure 3c). Percent moss cover, which is strongly influenced by environmental moisture, rapidly increased above ~893 m (±34 m [*SE*]).

This evidence suggests that the climatological cloud base in this region may be between 850 and 900 m, consistent with previous studies in the Appalachian Mountains (Markus, Bailey, Stewart, & Samson, 1991; Richardson et al., 2003). For the 1980–2012 DAYMET data, we found that relative humidity showed a positive relationship with latitude (Pearson's correlation coefficient, *r* = 0.30, *p* = 0.03). This relationship between relative humidity and latitude in the region of interest does not depend on the chosen averaging period (for example, for 2003–2012, *r* = 0.30, *p* = 0.04; for 2008–2012, *r* = 0.25, *p* = 0.08; for 2011–12, *r* = 0.24, *p* = 0.09). We therefore conclude that the relative humidity is typically higher around Stony Man than it is around Hawksbill, corresponding with a lower limit to the CBH on Stony Man.

Predictions of the future change in CBH were estimated under regional climate change forecasts from the suite of NARCCAP regional climate models. The smallest temperature increase results in an increase in mean CBH for the region of ~2.4 m per decade, while the largest temperature increase yields a CBH increase of ~4.1 m per decade.

## 4 DISCUSSION

Previous evidence has suggested that for vertebrates, and ectotherms in particular, species’ interactions are more important than abiotic factors in setting elevational range limits (Cahill et al., 2014). However, conservation actions focused on biotic interactions may fail unless environmental conditions are suitable; thus, it is important to understand when and where a species range is principally controlled by competition or environmental gradients (Urban, Tewksbury, & Sheldon, 2012). Future extinction risk may result from different climate sensitivities, not from biotic interactions, although these factors are seldom considered simultaneously in a single analysis (Cahill et al., 2013). Using a modeling framework that directly accounts for the presence of the red‐backed salamander and accommodates heterogeneity in detecting either species, we find that climate, not competition, is a chief determinant of the lower elevational range limit of the endemic *P. shenandoah* salamander.

While it has been hypothesized that the persistence of *P. shenandoah* in the presence of competitive pressure from *P. cinereus* may be facilitated by the presence of dew or fog (Jaeger, 1971a), we show here that the lower elevational range boundary itself is directly influenced by the presence of clouds. Our data confirm that the presence of clouds creates suitable conditions for the presence of *P. shenandoah* either directly from more frequent high humidity or indirectly through microhabitat, by the presence of moss. The presence of cloud moisture may similarly facilitate persistence of *P. shenandoah* in other areas of the range, where competition with *P. cinereus* may be relatively more important. The higher desiccation tolerance of *P. shenandoah* over *P. cinereus* (Jaeger, 1971a) would allow for persistence in these habitats during cloud‐free periods, at which time it is likely that the population retreats belowground.

Regional forecasts suggest increased warming in these high‐elevation habitats, so increases in CBHs observed across the Appalachians (Richardson et al., 2003) are expected to continue into the future, which may increase the extinction risk for *P. shenandoah*. With a maximum height of 1,235 m on Hawksbill and 1,220 m on Stony Man, even a small increase in CBH will result in a large reduction of the species’ total occupied extent. For the CBH to be stable, an increase in temperature must occur simultaneously with an increase in specific humidity (i.e., the mass of water vapor per mass of air). Predictions for precipitation are highly uncertain for the region but generally forecast decreases in summer (Fan, Bradley, & Rawlins, 2014). If the decrease in precipitation results in a decrease in specific humidity, which may be expected due to decreasing evapotranspiration rates, the rate of elevation change in the CBH will increase more rapidly than our estimates of 2.4–4.1 m per decade. Indeed, global analyses of elevation shifts suggest species have already increased their elevation range at median rates of 6.1 m (Parmesan & Yohe, 2003) to 11.0 m (Chen et al., 2011) per decade. As we find that the warm edge range limit is controlled by climate, extinction risk for *P. shenandoah* will be exacerbated under future climates. For *P. shenandoah* in particular, its recovery plan states that gradual erosion of the talus habitat, and subsequent competition by *P. cinereus*, is the dominant driver of extinction risk (Jaeger, 1970). Given the strong a priori expectation that competition is driving patterns of occupancy for *P. shenandoah* (Griffis & Jaeger, 1998; Jaeger, 1970, 1971b), we would have expected much higher support for conditional occupancy models. Instead, our data suggest that climate change is likely to be a much greater risk to *P. shenandoah*. This introduces a very interesting ecological question: Why does competition appear to limit the distribution of the species on other parts of the range edge, yet does not appear (via comparing the weight of evidence for conditional vs. unconditional models of occurrence) to determine the lower elevation limit? The difference may be the scale and location of the different studies. Griffis and Jaeger (1998) were focused on fine‐scale movement and microscale occupancy (tens of meters) at the lateral range edges, whereas our analysis focused on broad‐scale patterns of occupancy across the lower elevational boundary of the range (hundreds of meters). Thus, it may be that both climate and competition are acting to restrict the range of *P. shenandoah* and will each be important to consider in future predictions of extinction risk.

Cloud presence has been shown to be important for salamander species in other ecosystems as well. In the tropics, plethodontid salamanders reach highest diversity in high‐elevation cloud forests (Wake, Papenfuss, & Lynch, 1992; Wake & Vredenburg, 2008), and clouds affect distribution patterns for species in these communities (Wake et al., 1992). Clouds may provide moisture during critical dry periods, and changes in CBH may be one cause of population declines in tropical cloud forest communities (Pounds, Fogden, & Campbell, 1999; Rovito, Parra‐olea, Vásquez‐Almazán, Papenfuss, & Wake, 2009). We find that in the high‐elevation temperate forests of the Blue Ridge Mountains, the presence of clouds in high‐elevation pockets of talus habitat likewise creates distinct climatic refugia. Even though the climate of Shenandoah National Park has remained relatively stable in the last half‐century, future climate change is expected to alter the thermal and humidity environment in these high‐elevation habitats, increasing CBH elevations (Richardson et al., 2003).

Finally, we point out that current phenomenological models (e.g., climate envelope or ecological niche models) assume that identification of limiting variables on a species’ distribution is derived from unbiased species occurrence data, controlled by climate, and constant over time; application of these models typically contain some violation of one or more of these assumptions (Wiens & Bachelet, 2010; Yackulic et al., 2012). Reducing bias in identification of climatic variables that control the range limits of species is invaluable in predicting future range shifts and extinction risk, especially for high‐elevation species (Tingley & Beissinger, 2009). Further, unbiased inference is critical to identifying appropriate conservation and management actions at the appropriate scales (Wiens & Bachelet, 2010). Future extinction risk may be more sensitive to climate sensitivities, not biotic interactions. Conservation actions focused on the latter may fail unless environmental conditions remain suitable; thus, it is important to understand when and where a species range is controlled by competition or environmental gradients (Urban et al., 2012).

## CONFLICT OF INTEREST

None declared.

## AUTHOR CONTRIBUTIONS

EHCG, ABB, and JW designed research; EHCG, ABB, SFJDW, and TRL performed research; EHCG, SFJDW, and TRL analyzed data; all authors wrote the manuscript.
